# Impact of Oxysterols on Cell Death, Proliferation, and Differentiation Induction: Current Status

**DOI:** 10.3390/cells10092301

**Published:** 2021-09-03

**Authors:** Fábio Alessandro de Freitas, Débora Levy, Amira Zarrouk, Gérard Lizard, Sérgio Paulo Bydlowski

**Affiliations:** 1 Lipids, Oxidation and Cell Biology Team, Laboratory of Immunology (LIM19), Heart Institute (InCor), Faculdade de Medicina, Universidade de São Paulo, São Paulo 05403-900, Brazild.levy@hc.fm.usp.br (D.L.); 2 Faculty of Medicine, University of Monastir, LR12ES05, Lab-NAFS ‘Nutrition—Functional Food & Vascular Health’, Monastir, Tunisia & Faculty of Medicine, University of Sousse, Sousse 5000, Tunisia; zarroukamira@gmail.com; 3 Team ‘Biochemistry of the Peroxisome, Inflammation and Lipid Metabolism’ EA, University of Bourgogne Franche-Comté, Institut National de la Santé et de la Recherche Médicale—Inserm, 7270 Dijon, France; gerard.lizard@u-bourgogne.fr; 4 National Institute of Science and Technology in Regenerative Medicine (INCT-Regenera), CNPq, Rio de Janeiro 21941-902, Brazil

**Keywords:** apoptosis, autophagy, cell death, differentiation, oxysterols, mesenchymal stem cells, oxiapoptophagy

## Abstract

Oxysterols are oxidized derivatives of cholesterol produced by enzymatic activity or non-enzymatic pathways (auto-oxidation). The oxidation processes lead to the synthesis of about 60 different oxysterols. Several oxysterols have physiological, pathophysiological, and pharmacological activities. The effects of oxysterols on cell death processes, especially apoptosis, autophagy, necrosis, and oxiapoptophagy, as well as their action on cell proliferation, are reviewed here. These effects, also observed in several cancer cell lines, could potentially be useful in cancer treatment. The effects of oxysterols on cell differentiation are also described. Among them, the properties of stimulating the osteogenic differentiation of mesenchymal stem cells while inhibiting adipogenic differentiation may be useful in regenerative medicine.

## 1. Introduction

Cholesterol (cholest-5-en-3β-ol) is a major sterol (steroidal lipid) present in mammalian cells [1]. It is an important cell membrane compound with crucial roles in cell growth and proliferation. Cholesterol is involved in membrane biogenesis and function, controlling its physical properties such as fluidity and curvature [2,3,4]. In addition to this structural role, cholesterol also has several other functions: it is a precursor to bile acids, to vitamin D, and to a variety of steroid hormones [2]; it is involved in several intracellular signal transduction processes [5]; it regulates protein function [6]; it participates in myelin formation [7]; it acts as ligands to nuclear receptors and to G protein-coupled receptors (GPCRs) [8,9]. Cholesterol is very susceptible to oxidation [10,11,12], which favors the formation of derivatives called oxysterols [2,11,13], which can be found in low concentrations in the organism (nanomolar range in the plasma of healthy subjects) [14,15].

## 2. Oxysterols

Oxysterols are a large family of 27-carbon oxidized derivatives of cholesterol [16]. They are endogenously produced in vivo by a variety of cells via enzymatic activity, auto-oxidation (radical processes), or both [2,13,17,18]. Several major oxysterols arise as intermediates in the pathways converting cholesterol to bile acids or steroid hormones [19,20,21]. As a result of cholesterol oxidation, polar groups (hydroxy, keto, hydroperoxy, epoxy, or carboxyl) are added to the cholesterol molecule. In addition, oxysterols are also present in the diet. Phytosterols, which are oxygenated forms of plant sterols and some cholesterol precursors, can also originate oxysterols [22,23,24,25]. 

**Figure 1:**
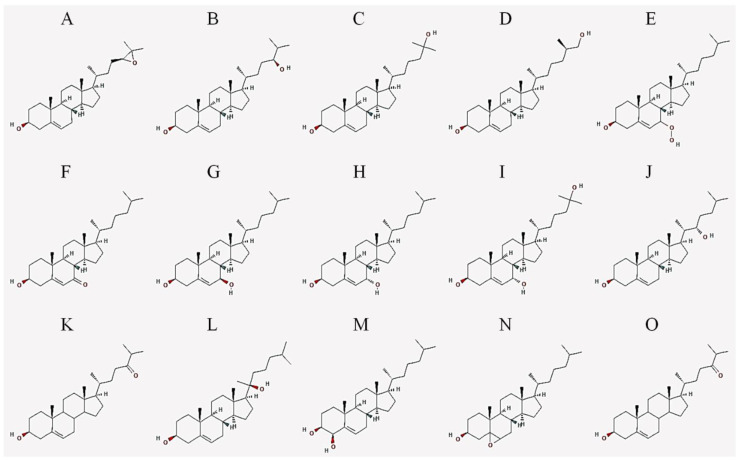
Oxysterol 2D molecules representations. (**A**) 24(S),25-epoxycholesterol; (**B**) 24(S)-hydroxycholesterol; (**C**) 25-hydroxycholesterol; (**D**) 27-hydroxycholesterol; (**E**) 7-hydroperoxycholesterol; (**F**) 7-ketocholesterol; (**G**) 7β-hydroxycholesterol; (**H**) 7α-hydroxycholesterol; (**I**) 7α,25-dihydroxycholesterol; (**J**) 22(S)-hydroxycholesterol; (**K**) 24-oxocholesterol; (**L**) 20(S)-hydroxycholesterol; (**M**) 4β-hydroxycholesterol; and (**N**) 5,6-epoxycholesterol; (**O**) 24-oxocholesterol. Source: PubChem; URL: https://pubchem.ncbi.nlm.nih.gov; last accessed, 5 August 2021.

Figure 1 shows several oxysterol derivatives. Table 1 lists some oxysterols with their common names, as well as their names according to the International Union of Pure and Applied Chemistry (IUPAC). 

In the enzymatic pathways, oxysterols are formed by the action of several specific enzymes. There are three groups of enzymes associated with oxysterol metabolism: oxidoreductases (e.g., cytochrome P450, cholesterol hydroxylase, hydroxysteroid dehydrogenases, and squalene epoxidase); hydrolases (e.g., cholesterol epoxide hydrolase, and cholesterol esterase); and transferases (e.g., hydroxysteroid sulfotransferases, acyl-CoA cholesterol transferase, and lecithin-cholesterol acyltransferase) [10,13]. Oxysterols generated by enzymatic processes commonly have oxidized side chains [10,26]. Interestingly, some genetic alterations of these enzymes, involved in oxysterol formation, have been associated with some types of cancer: *CYP7A1* gene polymorphism -204A > C, rs3808607 has been associated with colorectal cancer [27] while polymorphisms -204 A > C, rs3808607, and -469 T > C, rs3824260, have been associated with increased gallbladder cancer risk [28]; CYP3A4*1B gene polymorphism rs2740574 A > G has been associated with an increase in prostate cancer among African populations [29]; decreased gene expression of 11β-HSD1 and overexpression of 11β-HSD2 have been described in breast cancer [30,31].

Endogenous oxysterols are commonly produced by non-enzymatic mechanisms (auto-oxidation of cholesterol molecules), with oxidation taking place in the sterol ring [6]. Generally, reactive oxygen species (ROS) are involved. Molecules such as singlet oxygen, hydrogen peroxide, hydroxyl radical, and ozone, oxidize the cholesterol molecule in lipoproteins and cell membranes, but also in food [10,32]. This reaction can promote the abstraction of an allylic hydrogen atom at C-7, and this carbon atom can easily react with molecular oxygen, forming a cholesterol peroxyl radical (COO-) [26].

**Figure 2:**
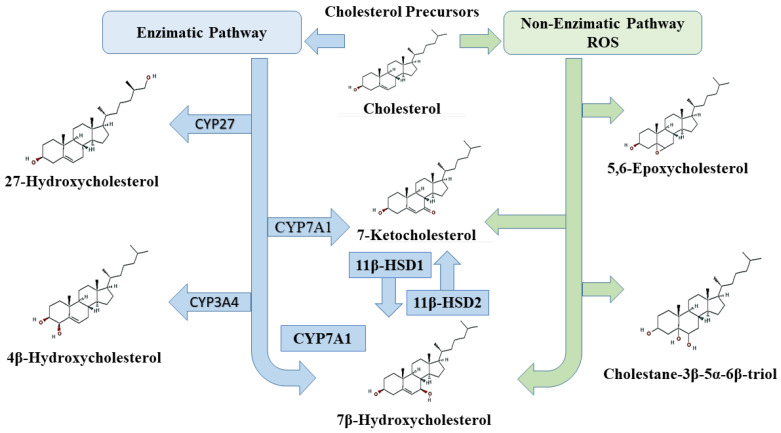
Schematic representation of synthesis of some oxysterols via enzymatic and non-enzymatic pathways.

Some oxysterols such as 7-ketocholesterol (7-KC), 7β-HC, 25-HC, and 7α-HC can be generated by both pathways [32]. A schematic representation of enzymatic and non-enzymatic pathways is shown in Figure 2.

Oxysterols can also be formed in food during heating or prolonged storage of cholesterol-containing products. High oxysterol levels can be found in several foods, such as powdered milk, cheese, egg products, and meat. Some oxysterols can also be formed from cholesterol in the stomach where all the conditions are in place to promote cholesterol oxidation: the acidic pH, and the presence of oxygen, iron ions, or metmyoglobin from dietary origin, all produce a highly pro-oxidizing environment [33]. Humans can absorb oxysterols from food into the bloodstream, where they are rapidly cleared from the plasma and re-distributed to different tissues of the body [34]. Tissues and organs may take up oxysterols from the plasma several times faster than cholesterol [35]. However, oxysterols are present in mammalian tissues at very low concentrations [19]. The main oxysterols commonly identified in human plasma include 7α-HC, 24(S)-HC, 4α-HC, and 4β-HC [36].

Oxysterols have been identified as a class of highly relevant signaling molecules that act in several human biological systems [1,22,35,37,38,39]. They play important roles in physiological and pathological processes, including cholesterol homeostasis, immune system regulation, platelet aggregation, inflammation, cell differentiation and proliferation, osteoporosis, age-related macular degeneration, atherosclerosis, cardiovascular disease, neurodegenerative diseases such as Alzheimer’s disease, Parkinson’s disease, multiple sclerosis, and in the development and progression of some cancers [40,41,42].

Oxysterols can activate or inhibit several cellular signaling pathways [43] acting on cellular receptors, including nuclear receptors, a large superfamily of 48 ligand-inducible transcription factors. These proteins act as intracellular receptors that bind to lipophilic ligands capable of crossing the plasma membrane [44]. Of the nuclear receptors, the liver X receptors (LXRs) α and β are members of the nuclear receptor superfamily that regulate cholesterol homeostasis [43,45]. Oxysterols act as ligands of LXRα (NR1H3) and LXRβ (NR1H2) regulating the transcription of specific genes. LXRα is expressed primarily in the liver, intestine, adipose tissue, macrophages, and adrenal gland, whereas LXRβ is expressed in many cell types [17]. Upon binding to oxysterols, these transcription factors form obligate LXR–retinoid X receptor heterodimers, which interact with DNA to regulate the transcription of target genes [46]. Many LXR target genes are involved in cholesterol and fatty acid metabolism, including ABCA1, ABCG1, SREBP-1c, and fatty acid synthase. Other targets, such as AIM/SPa, are involved in the regulation of apoptosis and innate immune responses [45].

In addition, the role of oxysterols in cell death and differentiation processes is gaining attention. Many studies have shown that oxysterols exhibit cytotoxicity in several cells, including vascular cells (smooth muscle cells, vascular endothelial cells, and fibroblast) [10,41,47,48] and nerve cells (glial and microglial cells, and neurons) [41,48].

## 3. Oxysterols and Cell Death

Cell death is an important process for maintaining organism homeostasis. It eliminates old and injured cells, arising after cell damage or triggered by specific signaling. Cell death can be described broadly as an irreversible degeneration of vital cellular functions, ending with a loss of cellular integrity. This loss of integrity can be characterized by fragmentation or the permanent permeabilization of the plasma membrane [49]. This process is highly organized and crucial to normal physiological processes, such as embryonic development and tissue renewal. Cell death is also implicated in several other mechanisms, including the maintenance of epithelial barrier function, adaptative immune responses, recycling of biologic macromolecules, intracellular signaling, and preservation of genomic integrity [50]. However, it can also be involved in several pathological responses, such as cancer, cell injury and response to infectious pathogens.

A Nomenclature Committee on Cell Death (NCCD) was created to formulate guidelines for the definition and interpretation of cell death, taking into consideration its morphological, biochemical, and functional characteristics. With new analytical methodologies, novel mechanisms of cell death and new cell death pathways have been described [49].

The first reports on oxysterol-induced toxicity came out in the 1970s. Interest has grown since that time, with studies aiming to elucidate the mechanism that involves oxysterols and their cytotoxic action [51]. Because of the large number of ways that cholesterol can undergo oxidation, more than 60 different oxysterols have been reported. Despite this variety, only some of these oxysterols have cytotoxic properties. Some of them are, in fact, potent inducers of cell death [52]. Oxysterols can influence cancer progression and can act either as oncometabolites or as tumor suppressors based on the tumor microenvironment [53].

**Figure 3:**
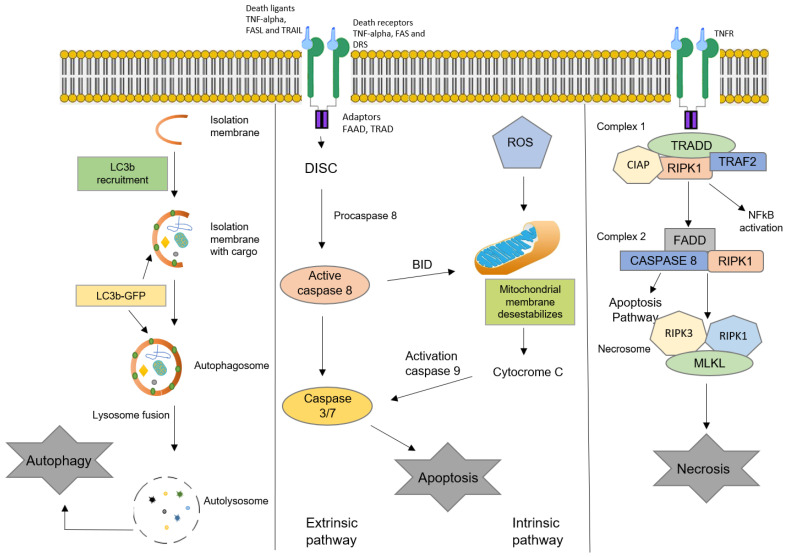
Schematic representation of the three main types of cell death: autophagy, apoptosis, and necrosis. TNF (tumor necrosis factor); FAS (CD95); FAS-L (CD95-ligand); TRAIL (TNF-related apoptosis-inducing ligand); TNFR (TNF receptor); ROS (reactive oxygen species); FADD (Fas-associated death domain); TRADD (TNFR-associated death domain); RIPK (receptor-interacting protein kinase); MLKL (mixed lineage kinase domain-like protein); DISC (death-inducing signaling complex).

Generally, oxysterols are pro-apoptotic or pro-autophagic, but in high concentrations they can induce necrosis in some cell lineages [54]. However, the effects that oxysterols exert on cells are dependent on the cell lineage and the type of oxysterol, and on its concentration [2,19]. The main types of cell death promoted by oxysterols (apoptosis, autophagy, and necrosis) are described below and are schematically shown in Figure 3 [55].

### 3.1. Apoptosis

Apoptosis (from a Greek word meaning “falling off”, like leaves from a tree) is a form of programmed cell death [56,57,58] involving a mechanism of self-inflicted death encoded in the genetic material of cells [50]. Kerr et al. first described apoptosis, also known as programmed cell death, in the early 1970s [16,57,59,60]. Since its discovery, apoptosis has been one of the most studied biological processes of cellular death [61]. Unlike necrosis, a cell triggers apoptosis and follows a course toward death upon the detection of certain stimuli [61]. Apoptosis is associated with: (a) activation of mitochondrial permeability, with loss of transmembrane mitochondrial potential and release of cytochrome c into the cytosol; (b) activation of caspases; (c) condensation of chromatin (pyknosis); (d) endonuclease activation followed by internucleosomal DNA cleavage (multiple of 180–200 pairs of bases); (e) segregation of nucleoli; (f) nucleus fragmentation and/or condensation; and (g) blebbing of plasma membrane associated with the formation of apoptotic bodies [59,61].

Two apoptosis pathways have been described: the intrinsic and extrinsic. Intrinsic pathway signaling is linked to mitochondria function (mitochondrial pathway) [62,63]. ROS, in particular hydroxyl radical (OH^−^), hydrogen peroxide (H_2_O_2_), and superoxide anion (O_2_^−^), are toxic byproducts of oxidative phosphorylation. ROS is involved in the oxidative damage of mitochondrial lipids, DNA, and proteins, making mitochondria even more prone to ROS production. In turn, damaged mitochondria release high levels of Ca^+^ and cytochrome c into the cytosol, triggering apoptosis [64].

The extrinsic pathway (death receptor pathway) is triggered by cell surface receptors that are stimulated by extracellular death-inducing signaling [62,63]. It has been proposed [62] that in the extrinsic pathway, extracellular signals or stress lead to the prompt release of ligands such as tumor necrosis factor (TNF), CD95-ligand (CD95-L or Fas-L), TNF-related apoptosis-inducing ligand (TRAIL or Apo2-L), and TNF-like ligand 1A (TL1A), which can bind to the death receptors. These death receptors are members of the TNF family and include TNF receptor-1 (TNFR-1), Fas, Apo-1, and TRAIL receptors (TRAIL-Rs). This receptor–ligand binding leads to the recruitment of the procaspase-8 enzyme to the death-inducing signaling complex (DISC). At the cytoplasmic end of the death receptor, adaptor proteins, such as the Fas-associated death domain (FADD) or TNFR-associated death domain (TRADD), are recruited. This recruitment results in the dimerization and activation of caspase-10 and caspase-8 monomers and, ultimately, in the dimerization of caspase-8 and the subsequent activation of the effector caspases-3/6/7 [62].

### 3.2. Autophagy

Autophagy is a normal cellular process involving intracellular degradation of cytoplasmic components, including organelles, proteins, and lipids. These components are sequestered inside vesicles (autophagosomes), formed by a double membrane, and delivered to the lysosome for degradation [65,66,67], providing substrates and energy [67].

The term autophagy was first used by Christian De Duve, the discoverer of lysosomes and peroxisomes and the first scientist to conduct experiments to demonstrate the biochemical involvement of the lysosomes in this process [68]. In 2016, Yoshinori Ohsumi received the Nobel Prize for Physiology and Medicine for his discovery of the mechanisms of autophagy using yeast as a model. One of his most important findings was the role of the ubiquitin-like proteins (UBLs) Atg5, Atg12, and Atg8 in the formation of the double-membrane vesicle (autophagosome), which is the functional unit of autophagy [66].

In summary, two UBL systems are involved in autophagosome formation. The first stage is the formation of a covalent link between Atg12 and Atg5 through to Atg7 (E1-like enzyme) and Atg10 (E2-like enzyme). The second stage is the formation of a covalent link between the microtubule-associated protein light chain-3 (LC3) and phosphatidylethanolamine (PE) through Atg7 and Atg3 (E2-like enzyme). The autophagosome then fuses with the lysosome, causing out the disintegration of the inner autophagosomal membrane and degradation of autophagosome content by lysosomal enzymes. This process provides amino acids, free fatty acids, and other products that cells can use for other purposes [69].

Autophagy is involved in several pathophysiological processes such as cancer, neurodegenerative disease, aging, autoimmune diseases (such as Crohn’s disease and rheumatoid arthritis), heart disease, and infection, and can be a mechanism of caspase and apoptosis-independent cell death. In fact, the ability of autophagy to modulate cell death makes it a therapeutic target (through either up- or down-regulation) for several conditions such as cancer and neurodegenerative diseases [70].

However, autophagy can be triggered in any cell stimulated by stress or nutrient deprivation. Autophagy can be activated when cellular components are damaged, providing cells with molecular raw materials and energy [70,71,72]. Thus, autophagy can be considered to be a kind of internal quality control of organelles and proteins in cell machinery, with an important role as a survival mechanism [67]. Considering all the cellular processes involved, autophagy can exhibit pro-death or pro-survival functions. The pro-survival functions of autophagy are related to its support for cells in dealing with stress, clearing damaged proteins, organelles, pathogens, or aggregates, or providing the cell with energy and anabolic products during starvation [70].

### 3.3. Necrosis

Necrosis is an uncontrolled mechanism of cell death that occurs in response to extreme cellular injuries and trauma [50,73,74]. Necrosis can be induced by physical or environmental factors such as ischemia [50,73], infections, toxins, mechanical trauma, or thermal damage from extremely high or low temperatures [74]. Some tissues during inflammation or infection processes can secrete cytokines that can initiate necrosis pathways [75]. It could be considered as a failure of the cellular homeostatic process [50,73], leading the cell to premature death by autolysis [74]. The necrosis process involves disruption of the plasma membrane, followed by the release of cell contents into the extracellular space, triggering the recruitment of neutrophils, macrophages, and other elements of an acute inflammatory response [73,74]. Cells recruited in the necrosis process eliminate the dead cells and their products by phagocytosis [74].

Some studies have demonstrated that necrosis can apparently be a programmed and regulated form of cell death. Different types of necrosis have been identified, including programmed necrosis such as necroptosis, pyroptosis [56], ferroptosis (iron-dependent cell death) [56,76], mitotic catastrophe, and autophagic cell death [56].

### 3.4. Main Oxysterols Involved in Cell Death

Several, but not all, oxysterols promote cell death. Table 2 summarizes the main oxysterols that are involved in this process.

#### 3.4.1. 7-Ketocholesterol (7-KC)

7-KC is one of the most studied oxysterols and has been described as one of the most toxic and predominant components of oxidized low-density lipoprotein (oxLDL) [52,90,91]. 7-KC has been involved in the development of major age-related diseases such as macular degeneration, cataracts, atherosclerosis, and cardiovascular diseases, Alzheimer’s disease (including vascular dementia), and multiple sclerosis [88]. The mechanism with which 7-KC causes cell death in major age-related diseases is linked with the activation of several kinase signaling pathways via multiple transcription factors inducing cytokines and intracellular effectors [41].

7-KC can induce cell death by apoptosis in cancer cells such as melanoma, breast cancer, and colon carcinoma [2,52,81,88,92,93], where sonic hedgehog and LXRα pathways might be involved [94]. 7-KC was also shown to be effective in the induction of apoptosis in a chronic myeloid leukemia cell line exhibiting a multidrug resistance phenotype (MDR) [82]. 7-KC is also a potent inducer of apoptosis in normal cells, such as endothelial cells, fibroblasts, and mesenchymal stem cells, at least in part by direct action on mitochondria [2,47]. Interestingly, autophagy was shown to be up-regulated as a cellular protective response to 7-KC cell death induction in human aortic smooth muscle cells [83].

7-KC has also been described to promote the induction of oxiapoptophagy, a complex mode of cell death characterized by ROS overproduction (“oxi-”), apoptosis induction, (“-apopto”), and autophagy (“-phagy”), in several cells, such as human monocytic U937 cells, mesenchymal stem cells, murine oligodendrocyte 158N cells, microglial BV-2 cells, and neuronal N2a cells [41,52,77,79,80,92]. In oxiapoptophagy, oxidative stress is associated with the overproduction of reactive oxygen species, increased antioxidant enzyme activities, lipid peroxidation, and protein carbonylation [52,77,79,80,95].

Some studies have demonstrated that 7-KC also induces apoptosis via phospholipidosis [78]. Phospholipidosis is a lysosomal storage disorder characterized by the excess accumulation of phospholipids in cells, with an ultrastructural appearance of multilamellar cytoplasmic inclusions (called “myelin figures”), which are predominantly lysosomal in origin [54]. The relationship between phospholipolysis and cell toxicity is not totally understood, but phospholipolysis might be part of a cell death process involving caspase activation, leading cells to apoptosis [54]. Phospholipidosis, characterized by the presence of “myelin figures” in large vacuoles, could correspond to reticulophagy [96].

#### 3.4.2. 7β-Hydroxycholesterol (7β-HC)

Like 7-KC, 7β-HC is a potent inducer of apoptosis and oxiapoptophagy in tumor and normal cells [52]. 7β-HC has cytotoxic action at low concentrations (µM) in both normal and tumor cell lines in culture. It can also reduce the growth of experimental murine transplanted tumors [16].

7β-HC can induce cell death in the 158N murine oligodendrocyte lineage [51]. This death is associated with oxidative stress and fatty acid metabolism dysfunction. The cytotoxic effects are related to cell death by mitochondrial dysfunction and the enhancement of glutathione peroxidase (GPx) and superoxide dismutase (SOD) activities. These antioxidant enzyme activities are considered a cellular reaction against ROS overproduction and overproduction of lipid peroxidation products (malondialdehyde and conjugated dienes). In addition, 7β-HC has also been described to be a cytotoxic agent that suppresses the growth of some types of head and neck cancer by its action on cyclooxygenase-2 [84]. It has also been shown that 7β-HC acts on Fas death receptor- Fas ligands, and that this interaction starts the caspase cascade that leads to the apoptosis pathway [85].

Interestingly, it has been described that 7β-HC can induce a non-apoptotic mode of cell death associated with pro-survival autophagy in C6 glioma cells [86]. Moreover, like 7-KC, 7β-HCs is also a major component of oxidized LDL and has been associated with the development of atherosclerosis, cardiovascular diseases, and cataracts [88].

#### 3.4.3. Cholestane-3β-5α-6β-triol

Cholestane-3β-5α-6β-triol is one of the most abundant and active oxysterols [87,97]. Like 7-KC, it was shown to induce cell death by apoptosis in cancer cell lineages such as melanoma, breast cancer, and prostate cancer, as well as in normal cell lineages such as fibroblasts and endothelial cells [2].

Nevertheless, cholestane-3β-5α-6β-triol has been shown to have neuroprotection effects in vitro against glutamate-induced neurotoxicity via negative modulation of NMDA receptors [97]. Moreover, cholestane-3β-5α-6β-triol significantly decreases neuronal injury after spinal cord ischemia in rabbits and transient focal cerebral ischemia in rats [97].

#### 3.4.4. Effects of Other Oxysterols

5α-cholestane-3β,6β-diol was shown to induce apoptosis in melanoma B16-F10 and breast cancer MDA-MB-231 cell lineages and in normal cell lineages (endothelial HUV-EC-C and fibroblast LL-24) [2].

20(S)-hydroxycholesterol (20(S)-HC) can be enzymatically produced in the endoplasmic reticulum (ER) of neurons, playing an important role in the cholesterol homeostasis of the brain. However, it also promotes neurotoxicity when esterified in the ER by acyl-CoA:cholesterol acyltransferase 1 (ACAT1) [98].

7,25-dihydroxycholesterol (7,25-DHC) and 25-hydroxycholesterol (25-HC) have cytotoxic action at low concentrations, acting in both normal and tumor cultured cell lines, mainly in lymphocytes, and can reduce the growth of experimental murine transplanted tumors [16].

24-hydroxycholesterol (24-HC) has cytotoxic effects on neuronal cells and has some influence on the development of neurodegenerative diseases [89]. It has been shown that 24-HC is responsible for the induction of necroptosis in SH-Sy5Y cells and can induce both apoptosis and necroptosis in lymphoma cells [89].

## 4. Oxysterols and Cell Proliferation

Oxysterols are also involved in cell proliferation. For instance, in adipose tissue-derived stem cells (SCs), 5α-cholestane-3β,6β-diol inhibits cell proliferation but not cell cycle progression [2].

The first reports on the antiproliferative and anti-cancer activities of oxysterols came out in the 1970s. Oxysterols can interfere in the proliferation of several cancer cell types (glioblastoma, leukemia, colon, breast and prostate cancer). However, they have little or no effect on senescent cells [99]. They can interfere with cholesterol homeostasis, acting on LXRs and 3-hydroxy-3-methylglutaryl CoA reductase, as well as on other pathways, such as extracellular signal-regulated kinase (ERK), hedgehog (Hh), and Wnt, which are all associated with proliferation [99]. An increase in cholesterol levels in cancer cells elevates oxysterol production, consequently controlling cholesterol homeostasis in LXR-dependent or LXR-independent pathways [53]. Cholesterol efflux involving LXR arises via the induction of ATP-binding cassette (ABC) transporters [100], which are in the plasma and cell organelles’ membranes [101].

In fact, some strategies using oxysterols in experimental cancer treatment include receptor targets such as LXRs, which represent an additional putative mechanism for oxysterol-mediated pro-apoptotic effects. LXR agonists were described to promote cholesterol efflux in some human cancer cell lines, leading to the inhibition of cell proliferation and stimulation of apoptosis [100]. LXRs can inhibit cell proliferation in several cancer cells: colorectal, ovarian, breast, prostate, and gallbladder cancers, in addition to glioblastoma, melanoma, and leukemia. This action is possible because LXRs are lipid-sensing receptors that modulate cholesterol metabolism. As noted, the anti-proliferative action of LXRs is connected to reduced cellular cholesterol levels, stressing cancer cells, which have high cholesterol needs because of their high proliferation rates. LXR-mediated expression is decreased consistently in high-proliferation cell lines and cancer cells, and LXR ligands can inhibit this proliferation, making LXRs potential targets for cancer therapy [22]. Taking these findings together, oxysterols show considerable promise as immunosuppressants or anticancer agents [16].

25-hydroxycholesterol (25-HC) has been associated with cancer cell invasion and migration by modulating the expression of LXRs in lung, breast, and endometrial cancers [53,78]. It also activates estrogen receptors (ERs), promoting breast cancer cell proliferation [44,102].

26-hydroxycholesterol (26-HC) is synthesized by a mitochondrial P-450 enzyme, cytochrome *P-450 27A1* (*CYP27A1*), a gene encoding a cytochrome P-450 oxidase, [103] and is associated with breast cancer etiology [78], acting in immune cells favoring breast cancer metastasis. Some evidence indicates that high-fat diets can increase metastasis risk in breast cancer, linked to 26-HC [104]. Animal studies suggest that the inhibition of CYP27A1, which converts cholesterol to 26-HC, can reduce cancer risk. Therefore, the inhibition of CYP27A1 could be a promising target for preventing diseases related to this oxysterol [104].

27-hydroxycholesterol (27-HC), like 25-HC, is associated with effects on the invasive and migration properties of cancer cells by modulating LXR expression in lung, breast, and endometrial cancer [53,105,106]. 27-HC can affect the proliferation and viability of C6 glioma cells, increasing apoptosis and altering cholesterol homeostasis of this tumor [53]. 27-HC also acts on colon cancer cell lines with an anti-proliferative effect, independently of ERs and LXRs receptors [53].

Mammalian cells have two main subtypes of nuclear ERs: ERα (or NR3A1) and ERβ (or NR3A2) [44,102]. 27-HC is an oxysterol that can act as a selective ER modulator [44,102]. As approximately 60%–75% of all breast cancers are ERα+, the effect of 27-HC on breast cancer has been extensively studied in preclinical models [44,102]. CYP27A1 protein expression is associated with higher breast tumor grade, as shown above, while the expression of *CYP7B1* mRNA (a gene that encodes 7-α-hydroxylase) is associated with a better prognosis, indicating a clinically relevant role for 27-HC in breast cancer [44,102].

5,6-epoxycholesterols (5,6-ECs) are involved in the pharmacology of tamoxifen (an antitumor drug) [107]. 5,6-EC metabolites were described to have a role in the suppression or promotion of breast cancer [104].

Cholestane-3β-5α-6β-triol was shown to suppress the proliferation of human prostate cancer cells such as LNCaP CDXR-3 (androgen receptor-positive) and DU-145 and PC-3 (both androgen receptor-negative) [87]. It also inhibits colony formation of PC-3 and LNCaP CDXR-3 cells slows the growth of androgen-insensitive prostate cancer (DU-145 and PC-3) xenografts in nude mice, and causes G1 cell cycle arrest in LNCaP CDXR-3 and DU-145 cells [87].

## 5. Oxysterols and Cell Differentiation

Mesenchymal stem cells (MSCs) are multipotent cells, having the characteristics of self-renewal and the ability to differentiate into several cell types, such as osteoblasts, adipocytes, and chondrocytes. Due to these characteristics, MSCs have been used on a large scale in clinical research. Oxysterols can influence MSC differentiation [10], stimulating nuclear binding receptors, and activating differentiation pathways [47]. Table 3 summarizes the action of several oxysterols in cell differentiation processes.

20(S)-hydroxycholesterol (20(S)-HC) is the most potent oxysterol that stimulates osteogenic differentiation of MSCs. It also has an anti-adipogenic action on these cells [47]. 22(R)-hydroxycholesterol (22(R)-HC) significantly stimulates chondrogenesis in human adipose-derived mesenchymal stem cells; this effect involves upregulation on LXR signaling pathways [108]. The osteogenic effect of 20(S)-HC has been observed alone or in association with 22(S)-HC or 22(R)-HC, modulating critical pathways and preventing osteoporosis [88]. This ability to modulate the osteogenic differentiation of MSCs is due to the action of cyclooxygenase (COX)/phospholipase A2 (PLA2) and mitogen-activated protein kinase (MAPK), with activation of the Hh signaling pathway [88]. These oxysterols can also activate Wnt-related signaling pathways, regulating the proliferation and differentiation of osteoblasts during bone formation [88].

The association of 20(S)-HC with 22(S)-HC also promotes both in vitro osteogenic differentiation of stem cells from human periodontal ligament (involving a cross-reaction between the LXRs and the Hh signaling pathway), and in vivo alveolar bone regeneration in a rat model [116]. This association was also shown to induce the osteogenic differentiation of rat bone marrow mesenchymal stem cells in vitro [119]. Moreover, it also induces osteogenesis in a mouse embryonic stem cell line in vitro, involving upregulation of mitochondrial activity and an increase of Hh signaling target genes, Smo, and Gli1 expression [120].

Interestingly, the association of 20(S)-HC with simvastatin has a synergistic effect, enhancing osteogenic differentiation of rat bone marrow mesenchymal stem cells [111]. This association has also promoted bone regeneration in vivo in a rabbit model, via the stimulation of Raf/MEK/ERK signaling [111].

Oxy133 is a 20(S)-hydroxycholesterol oxysterol analog. It promotes in vitro osteogenic differentiation of the mouse multipotent bone marrow stem cell line M2-10B4, and in the embryonic fibroblast cell line C3H10T1/2, by activation of Hh pathway signaling. Oxi133 can also inhibit adipogenic differentiation in the C3H10T1/2 cell line in vitro and stimulates bone formation and spinal fusion in vivo in a rat model [122]. Oxysterol 49 is another semisynthetic oxysterol analogue that promotes osteogenic differentiation in vitro in primary rabbit mesenchymal stem cells by Hh pathway signaling [123].

5α-cholestane-3β-5α-6β-triol inhibits osteoblastic differentiation and promotes apoptosis of rat bone marrow cells [88]. It has been suggested that some oxysterols, such as 5α-cholestane-3β-5α-6β-triol and 5α-cholestane-3β,6β-diol, may contribute to osteoporosis [88].

27-hydroxycholesterol (27-HC) seems to be involved in osteoclastogenesis induction by regulating the miR-139/c-Fos pathway on RAW264.7 cells [113]. 27-HC was also described to inhibit osteoblast differentiation and activity on mouse primary calvarial (pre)osteoblast culture. This inhibition was related to 27-HC actions on ER and LXR receptors in osteoblasts [114]. In addition, 27-HC does not promote adipogenic differentiation. However, it stimulates the increase the in cell population that differentiates to adipocytes [124].

In addition, 27-HC is involved in the hyperplasia and increase in both the total number and M1-type macrophages of visceral white adipose mass in mice, favoring the increase of both body weight and adipose inflammatory gene expression by ERα, but not LXR receptors [124]. 27-HC induces the differentiation and activation of monocytes into mature dendritic cells. However, cyclosporin A can inhibit this process in human monocyte/macrophage THP-1 cells [125]. Moreover, dexamethasone can completely suppress the differentiation of THP-1 cells into mature dendritic cell phenotypes induced by 27-HC [112].

27-HC, in combination with 7α-HC, 7β-HC, 5α,6β-epoxycholesterol, 5β,6β-epoxycholesterol, cholestan-3β,5α,6β-triol, 7-oxo-cholesterol, and 25-hydroxycholesterol, was shown to stimulate human macrophage (M0) polarization to M2 immunomodulatory functional phenotype [118].

7-ketocholesterol decreases the viability of human adipose-derived stem cells, as well as interfere in the adipogenic differentiation of these cells by the sequential activation of WNT/β-catenin, p38-MAPK, ERK1/2, and JNK signaling pathways [117]. In addition, 7-KC inhibits the syncytialisation and differentiation of primary human placental trophoblast cells in vitro by LXR activation. The same effects are produced by 25-HC and 22(R)-HC [126].

25-hydroxycholesterol (25-HC) promotes the inhibition of adipogenesis in the mouse pluripotent cell line C3H10T1/2, suggesting a potential application in osteoporosis and obesity treatment. Interestingly, these actions are not mediated by hedgehog and LXR signaling [109]. High levels of 25-HC have been described as negatively involved in neuronal and muscular development in zebrafish [127].

Effects of other oxysterols. 24S,25-epoxycholesterol promotes midbrain dopaminergic neurogenesis in vivo in mouse models by the stimulation of both LXRα and LXRβ [115]. 7β,27-dihydroxycholesterol and 7α,27-dihydroxycholesterol are RAR-related orphan receptor gamma t (RORγt) ligands in both murine and human primary CD4+ T, promoting differentiation of these cells into IL-17–producing CD4+ Th17 T cells in vitro [121]. Cholest-4,6-dien-3-one induces epithelial-to-mesenchymal transition on human biliary tree stem/progenitor cells, while it impairs the differentiation of these cells in mature cholangiocytes and reduces their telomerase activity [110].

## 6. Conclusions

Several oxysterols can exhibit pathophysiological and pharmacological activities and may promote, at high levels, adverse effects in age-related diseases such as cardiovascular, neurodegenerative, and ocular diseases. In fact, several oxysterols are known to be inducers of cell death, including in several cancer cell lines, or even in vivo. The main cell death pathways affected by oxysterols are apoptosis, autophagy, necrosis, and oxiapoptophagy. Therefore, these characteristics have the potential to make oxysterols useful as cytostatic and cytotoxic adjuvants in cancer treatment.

In these different contexts, some oxysterols may act indirectly through lipid oxidation products such as 4-hydroxynonenal (4-HNE) which can be generated by lipid peroxidation as a consequence of the overproduction of ROS induced by some oxysterols, especially cytotoxic oxysterols [128,129]. Thus, 4-HNE, like other aldehydes, could contribute to disrupting cell signaling. This could be due in part to the consequences of protein carbonylation modifying certain signaling pathways as well as ligand-receptor interactions [130]. Moreover, depending on the concentration of 4-HNE, this aldehyde could also contribute directly or indirectly to inflammation and/or cell death [131].

On the other hand, on the basis of the ability of certain oxysterols to trigger differentiation, directly or indirectly, on different cell types, it is tempting to postulate that some oxysterols or some of their derivatives are of interest in regenerative medicine. In this regard, it cannot be excluded that 4-HNE may also be involved in oxysterol-induced cell differentiation [132].

## Figures and Tables

**Table 1:** Common names of the main oxysterols and their chemical nomenclature.

Abbreviation	Common Name	IUPAC Name
24,25-EC	24(S),25-epoxycholesterol	(3S,8S,9S,10R,13R,14S,17R)-17-[(2R)-4-[(2S)-3,3-dimethyloxiran-2-yl]butan-2-yl]-10,13-dimethyl-2,3,4,7,8,9,11,12,14,15,16,17-dodecahydro-1H-cyclopenta[a]phenanthren-3-ol
24-HC	24(S)-hydroxycholesterol	(3S,8S,9S,10R,13R,14S,17R)-17-[(2R,5S)-5-hydroxy-6-methylheptan-2-yl]-10,13-dimethyl-2,3,4,7,8,9,11,12,14,15,16,17-dodecahydro-1H-cyclopenta[a]phenanthren-3-ol
25-HC	25-hydroxycholesterol	(3S,8S,9S,10R,13R,14S,17R)-17-[(2R)-6-hydroxy-6-methylheptan-2-yl]-10,13-dimethyl-2,3,4,7,8,9,11,12,14,15,16,17-dodecahydro-1H-cyclopenta[a]phenanthren-3-ol
27-HC	27-hydroxycholesterol	(3S,8S,9S,10R,13R,14S,17R)-17-[(2R,6R)-7-hydroxy-6-methylheptan-2-yl]-10,13-dimethyl-2,3,4,7,8,9,11,12,14,15,16,17-dodecahydro-1H-cyclopenta[a]phenanthren-3-ol
7-OOHC	7-hydroperoxycholesterol	(3S,8S,9S,10R,13R,14S,17R)-7-hydroperoxy-10,13-dimethyl-17-[(2R)-6-methylheptan-2-yl]-2,3,4,7,8,9,11,12,14,15,16,17-dodecahydro-1H-cyclopenta[a]phenanthren-3-ol
7α-HC	7α-hydroxycholesterol	(3S,7S,8S,9S,10R,13R,14S,17R)-10,13-dimethyl-17-[(2R)-6-methylheptan-2-yl]-2,3,4,7,8,9,11,12,14,15,16,17-dodecahydro-1H-cyclopenta[a]phenanthrene-3,7-diol
7β-HC	7β-hydroxycholesterol	(3S,4R,8S,9S,10R,13R,14S,17R)-10,13-dimethyl-17-[(2R)-6-methylheptan-2-yl]-2,3,4,7,8,9,11,12,14,15,16,17-dodecahydro-1H-cyclopenta[a]phenanthrene-3,4-diol
7-KC	7-ketocholesterol	(3S,8S,9S,10R,13R,14S,17R)-3-hydroxy-10,13-dimethyl-17-[(2R)-6-methylheptan-2-yl]-1,2,3,4,8,9,11,12,14,15,16,17-dodecahydrocyclopenta[a]phenanthren-7-one
7α,25-DHC	7α,25-dihydroxycholesterol	(3S,7S,8S,9S,10R,13R,14S,17R)-17-[(2R)-6-hydroxy-6-methylheptan-2-yl]-10,13-dimethyl-2,3,4,7,8,9,11,12,14,15,16,17-dodecahydro-1H-cyclopenta[a]phenanthrene-3,7-diol
7β,27-DHC	7β,27-dihydroxycholesterol	(3S,7R,8S,9S,10R,13R,14S,17R)-17-[(2R)-7-hydroxy-6-methylheptan-2-yl]-10,13-dimethyl-2,3,4,7,8,9,11,12,14,15,16,17-dodecahydro-1H-cyclopenta[a]phenanthrene-3,7-diol
22-HC	22(S)-hydroxycholesterol	(3S,8S,9S,10R,13S,14S,17R)-17-[(2S,3S)-3-hydroxy-6-methylheptan-2-yl]-10,13-dimethyl-2,3,4,7,8,9,11,12,14,15,16,17-dodecahydro-1H-cyclopenta[a]phenanthren-3-ol
20-HC	20(S)-hydroxycholesterol	(3S,8S,9S,10R,13S,14S,17S)-17-[(2S)-2-hydroxy-6-methylheptan-2-yl]-10,13-dimethyl-2,3,4,7,8,9,11,12,14,15,16,17-dodecahydro-1H-cyclopenta[a]phenanthren-3-ol
4β-HC	4β-hydroxycholesterol	(3S,4R,8S,9S,10R,13R,14S,17R)-10,13-dimethyl-17-[(2R)-6-methylheptan-2-yl]-2,3,4,7,8,9,11,12,14,15,16,17-dodecahydro-1H-cyclopenta[a]phenanthrene-3,4-diol
7,25-DHC	7,25-dihydroxycholesterol	(3S,8S,9S,10R,13R,14S,17R)-17-[(2R)-6-hydroxy-6-methylheptan-2-yl]-10,13-dimethyl-2,3,4,7,8,9,11,12,14,15,16,17-dodecahydro-1H-cyclopenta[a]phenanthrene-3,7-diol
5,6-EC	5,6-epoxycholesterol	(1S,2R,5S,11S,12S,15R,16R)-2,16-dimethyl-15-[(2R)-6-methylheptan-2-yl]-8-oxapentacyclo[9.7.0.02,7.07,9.012,16]octadecan-5-ol
24-OXO	24-oxocholesterol	(6R)-6-[(3S,10R,13R,17R)-3-hydroxy-10,13-dimethyl-2,3,4,7,8,9,11,12,14,15,16,17-dodecahydro-1H-cyclopenta[a]phenanthren-17-yl]-2-methylheptan-3-one

Source: PubChem; URL: https://pubchem.ncbi.nlm.nih.gov; accessed on 5 August 2021.

**Table 2:** Oxysterols and cell death.

Oxysterol	Cell Death	Cell Lineage or Animal Model	References
7-ketocholesterol	Oxiapoptophagy	MSC *	[52]
		Human monocytic U937 cells	[77,78]
		Murine oligodendrocytes 158N cells	[79,80]
	Short-term apoptosis	MSC **	[47]
		Mus musculus skin melanoma cells (B16-F10)	[2,81]
		Human mammary gland/breast cells (MDA-MB-231)	[2]
		Human endothelial umbilical vein/vascular endothelium cells (HUV-EC-C)	[2]
	Apoptosis	Lucena	[82]
	Phospholipidosis	Human monocytic U937 cells	[54]
	Autophagy	Human aortic smooth muscle cells (HASMCs)	[83]
7β-hydroxycholesterol	Oxiapoptophagy	Human monocytic U937 cells	[77]
		Murine oligodendrocytes 158N cells	[80]
	Cell death associated with oxidative stress, and metabolic dysfunctions	Murine oligodendrocytes 158N cells	[51]
	Apoptosis	Human tongue squamous cell carcinoma (SCC9)	[84]
		Human tongue squamous cell carcinoma (SCC25)	[84]
		Human tongue squamous cell carcinoma (CAL27)	[84]
		Human pharynx squamous cell carcinoma FaDu	[84]
		Human monocytic U937 cells	[85]
	Cell death process associated with several characteristics of survival autophagy	C6 rat glioma cells	[86]
24(S)-hydroxycholesterol	Oxiapoptophagy	Murine oligodendrocytes 158N cells	[80]
Cholestane-3β-5α-6β-triol	Short-term apoptosis	Mus musculus skin melanoma cells (B16-F10)	[2]
		Human mammary gland/breast cells (MDA-MB-231)	[2]
		Human endothelial umbilical vein/vascular endothelium cells (HUV-EC-C)	[2]
		Human lung fibroblasts (LL 24)	[2]
	Apoptosis	Human prostate cancer cell line (PC-3)	[87]
		Human prostate cancer cell line (DU-145	[87]
		Human LNCaP CDXR-3	[87]
		Rat bone marrow cells	[88]
5α-cholestane-3β,6β-diol	Short-term apoptosis	Mus musculus skin melanoma cells (B16-F10)	[2]
		Human mammary gland/breast cells (MDA-MB-231)	[2]
		Human endothelial umbilical vein/vascular endothelium cells (HUV-EC-C)	[2]
		Human lung fibroblasts (LL 24)	[2]
25-hydroxycholesterol	Apoptosis	Human acute lymphoblastic leukemia T lymphoblast (CEM-C1)	[16]
		Human acute lymphoblastic leukemia T lymphoblast (CEM-C7)	[16]
		Murine thymocytes	[16]
27-hydroxycholesterol	Apoptosis	C6 glioma cells	[53]
Oxisterol mixture ***	Apoptosis	Human monocytic U937 cells	[78]
24-hydroxycholesterol	necroptosis-like	human neuroblastoma cells (SH-SY5Y)	[89]

* Human bone marrow mesenchymal stem cells from acute myeloid leukemia patients; ** Human adipose tissue mesenchymal stem cells; *** 7β-hydroxycholesterol, 7-ketocholesterol, 27-hydroxycholesterol, and 25-hydroxycholesterol.

**Table 3:** Oxysterols and cell differentiation.

Oxysterol	Differentiation	Cell Lineage or Animal Model	References
Cholestan-3β,5α,6β-triol	↓ Osteogenesis	Rat bone marrow cells	[88]
20(S)-hydroxycholesterol	↑ Osteogenesis	Bone marrow stromal cells	[10]
		Embryonic stem cells	[10]
	↓ Adipogenesis	Bone marrow stromal cells	[10]
22(R)-hydroxycholesterol	↑ Chondrogenesis	Human adipose-derived mesenchymal stem cells	[108]
25-hydroxycholesterol	↓ Adipogenesis	Mouse pluripotent cell line (C3H10T1/2)	[109]
Cholest-4,6-Dien-3-One	Epithelial-to-mesenchymal transition	Human biliary tree stem/progenitor cells	[110]
20(S)-hydroxycholesterol associated with simvastatin	↑ Osteogenesis	Rats bone marrow mesenchymal stem cells (BNCC100381)	[111]
27-Hydroxycholesterol	Differentiation and activation of monocytes into mature dendritic cell	Human monocytes cells from acute monocytic leukemia (THP-1)	[112]
	↑ Osteoclastogenesis	Mice macrophage cells (RAW264.7)	[113]
	↓ Osteoblastogenesis	Mice primary calvarial (pre)osteoblast	[114]
24(S),25-Epoxycholesterol	Midbrain dopaminergic neurogenesis	Mice model	[115]
22(S)-hydroxycholesterol and 20(S)-hydroxycholesterol combination	↑ in vitro osteogenesis	Human periodontal ligament stem cells	[116]
	↑ in vivo alveolar bone regeneration	Rat model	[116]
7-ketocholesterol	↓ Adipogenesis	Human adipose-derived stem cells	[117]
Oxysterol mixture *	Macrophage (M0) polarization to M2 immunomodulatory functional phenotype	Human macrophage	[118]
22(R)-hydroxycholesterol and 20(S) hydroxycholesterol combination	↑ Osteogenesis	Rats bone marrow mesenchymal stem cell	[119]
		Mouse embryonic stem cells line (D3)	[120]
7β,27-dihydroxycholesterol and 7α,27-dihydroxycholesterol	IL-17–producing CD4+ Th17 T cells	Murine and human primary CD4+ T cells	[121]
Oxy133	↑ Osteogenesis	Mouse multipotent bone marrow stromal cell (M2-10B4)	[122]
		Mouse embryonic fibroblast cell (C3H10T1/2)	[122]
	↓ Adipogenesis	Mouse embryonic fibroblast cell (C3H10T1/2)	[122]
	Stimulates bone formation and spinal fusion	In vivo rat model	[122]
Oxysterol 49	↑ Osteogenesis	Primary rabbit mesenchymal stem cells	[123]

↑ stimulation ↓ inhibition; * 7α-hydroxycholesterol (4%), 7β-hydroxycholesterol (10%), 5α,6β-epoxycholesterol (8%), 5β,6β-epoxycholesterol (22%), cholestan-3β,5α,6β-triol (6%), 7-oxo-cholesterol (21%), 25-hydroxycholesterol (1%), and 27-hydroxycholesterol (28%), in particular 27-hydroxycholesterol.
